# A report card approach to describe temporal and spatial trends in parameters for coastal seagrass habitats

**DOI:** 10.1038/s41598-023-29147-1

**Published:** 2023-02-09

**Authors:** Alex B. Carter, Rob Coles, Jessie C. Jarvis, Catherine V. Bryant, Timothy M. Smith, Michael A. Rasheed

**Affiliations:** 1 Centre for Tropical Water & Aquatic Ecosystem Research (TropWATER), James Cook University, Cairns, QLD 4870 Australia; 2 University of North Carolina Wilmington, Wilmington, USA

**Keywords:** Ecology, Environmental sciences

## Abstract

Report cards that are designed to monitor environmental trends have the potential to provide a powerful communication tool because they are easy to understand and accessible to the general public, scientists, managers and policy makers. Given this functionality, they are increasingly popular in marine ecosystem reporting. We describe a report card method for seagrass that incorporates spatial and temporal variability in three metrics—meadow area, species and biomass—developed using long-term (greater than 10 years) monitoring data. This framework summarises large amounts of spatially and temporally complex data to give a numeric score that provides reliable comparisons of seagrass condition in both persistent and naturally variable meadows. We provide an example of how this is applied to seagrass meadows in an industrial port in the Great Barrier Reef World Heritage Area of north-eastern Australia.

## Introduction

Marine and coastal ecosystems provide important services, including provision of food, disturbance regulation (e.g. floods and storms), biodiversity support and cultural services that include the recreational, spiritual and aesthetic^1^. Despite increased international focus on conserving and protecting these vital regions, they are increasingly under threat worldwide^2,3^. Tracking appropriate ecosystem and habitat parameters over appropriate spatial and temporal scales so that declines can be detected, halted and potentially reversed is therefore critical^4^. This requires an adaptive management framework that is well-informed, flexible and responsive^5^, with decision support tools that are scientifically robust, recent, relevant and easily understandable by a range of stakeholders^6,7^.

Report cards are a popular and effective decision support tool to integrate ecological monitoring information and to communicate visually environmental condition in relation to desired goals, in a way that engages a range of stakeholders and informs management actions^8^. The report card framework provides an organizing guide for assessing ecosystem condition, the factors affecting it, trends through time and information that can be used to achieve management goals^2^. This format distils complex information into easily understood grades and scores with standardized messages, while the quantitative metrics used to determine grades remove the subjectivity of expert judgements and the risk of shifting baselines. Despite report cards becoming a key communications strategy in so many ecosystem and environmental monitoring programs, organising and summarising data in a way that represents meaningful ecological change presents challenges. What superficially seems to be a simple process can be complex, including separating out different spatial scales and scales of variability, different habitats, different stress regimes, suitable indicators and metrics and determining when a system diverges from its desired state given the constraints of a particular environment^2,9^.

Seagrasses grow in shallow coastal waters making them susceptible to urban, industrial and agricultural runoff, coastal infrastructure, ports and shipping, dredging, water pollution, habitat loss, reclamation, overfishing, climate change and sea level rise^10,11^. They are highly sensitive to environmental disturbance, particularly declines in water quality such as excessive sediment and nutrient loads from riverine discharge and reductions in available light for photosynthesis^9,12–15^. This sensitivity and the fact that seagrasses are integrators of environmental condition over time makes seagrass an ideal indicator for monitoring marine environmental health^4,16–19^.

Seagrasses are one of the most extensive benthic marine plant habitats in the Great Barrier Reef World Heritage Area (GBRWHA) in north-east Australia, where they often form diverse, multispecies meadows^20^. They provide important ecosystem services that include substrate stabilization, filtering organic matter, recycling nitrogen, baffling wave and tidal energy, and providing food and shelter for commercially important fish, prawns and some of the world’s largest remaining populations of dugong (*Dugong dugon*) and green sea turtles (*Chelonia mydas*)^21–24^. Seagrass meadows also are a globally important carbon sink^25^.

Developing a report card for seagrass in this region presents a number of challenges, including: (1) high species diversity and the occurrence of multi-species communities with transitions defined by environmental conditions (e.g. benthic light, depth, tidal exposure, temperature); (2) the potential for large spatial and temporal variability in seagrass condition indicators in response to natural environmental variation and for those indicators to respond independently of each other to pressures and impacts; and (3) the desired state of indicators varying among different seagrass communities and meadows, or within similar communities growing under different environmental conditions^9,12,20^.

In developing a report card score that reliably synthesises the available data it is necessary to navigate a way through these complexities. In this paper, we address this by describing the steps and approach used in the development of an annual seagrass report card devised for Gladstone Harbour in Australia’s GBRWHA. We use the 2020 report card as an example and we describe the broader regional applications.

## Results

### Report card framework

**Figure 1:**
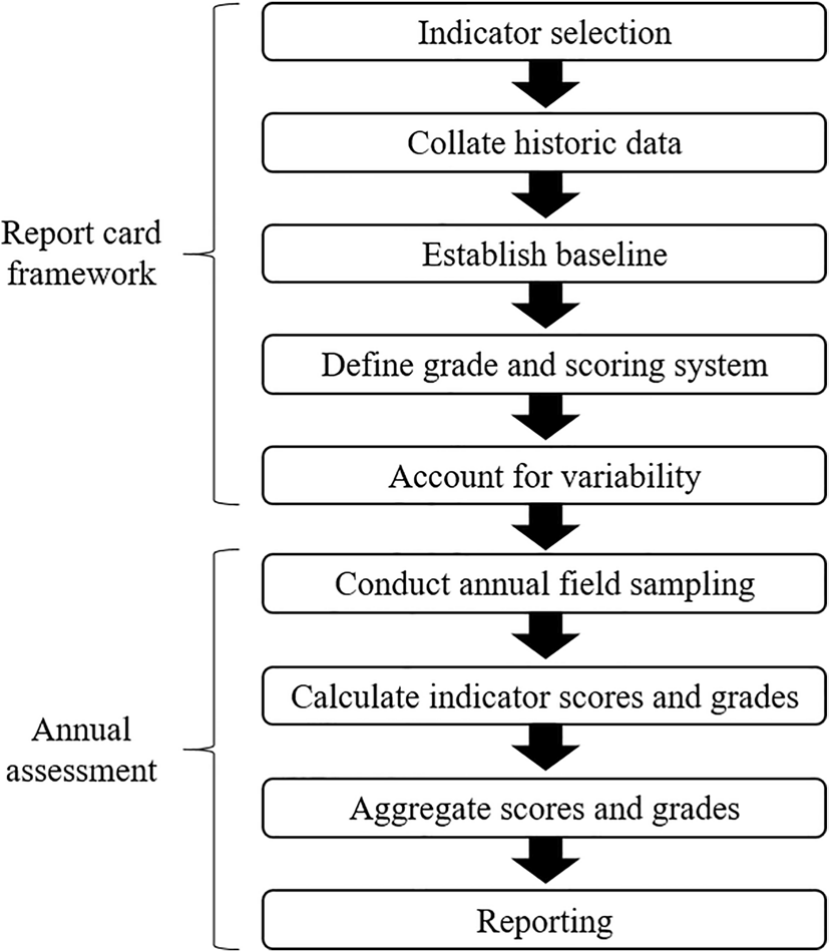
Flow chart to develop Gladstone Harbour seagrass grades and scores.

The first step in developing a seagrass report card as a component of the Gladstone Harbour report card was to design a framework against which annual monitoring data could be assessed. This included selecting seagrass condition indicators, collating historical monitoring data, establishing baselines, defining grades and a scoring system and defining grade thresholds that account for variability in historical data (Fig. 1). The second step was to assess seagrass condition within this framework using annual monitoring data. This included calculating meadow scores and grades and aggregating data where required (Fig. 1).

#### Indicators selected

The selection of appropriate indicators is challenging when developing a report card because the conclusions and management outcomes are dependent on the indicators selected^6^. Roca et al.^4^ identified 85 potential seagrass indicators with varying degrees of robustness. They encompass a broad range of traits, including the biochemical and physiological (e.g. C:N ratios, chloroplast density, dark respiration, photosynthesis rates), morphological and growth (e.g. leaf growth, root length, shoot size) and structural and demographic (e.g. above-ground biomass, below-ground biomass, cover, depth limit). For an annual report card we selected three seagrass indicators that capture change in Gladstone. These are metrics that for logistic reasons and due to the extensive scale of the harbour we could reasonably guarantee would always be measured as part of a continuing industry funded monitoring program.

- *Meadow area* as a measure of the extent of seagrass habitat;
- *Above-ground biomass* as a measure of the amount of seagrass available as food/ habitat within a meadow; and
- *Species composition* as a measure of a meadow’s diversity, and as an indicator of the meadow’s resilience^26^.

These indicators are also ideal because they reflect attributes considered essential to assessing seagrass community status and restoration success^27^ and their scientific interpretation is straightforward and meaningful to stakeholders and the public. They can be visually assessed rapidly using largely non-destructive methods that are cost effective and data are observer independent (see “Methods” section). For our purpose, baselines for these indicators were able to be defined using our existing monitoring data which included these metrics without the need for further research. They are reliable for an annual monitoring program because of their low short-term stochasticity (unlike flowering and fruiting) but are highly reactive to ecosystem health variability. Response times (degradation and recovery) to environmental change makes these indicators particularly suited to annual monitoring, with seagrass response times to environmental declines and recovery occurring over several years^18,28,29^. Above-ground biomass is a particularly robust indicator of any changing stressor levels including shading, nutrients and burial^4^ likely to be experienced by coastal seagrass in the GBRWHA.

#### Baselines established

**Figure 2:**
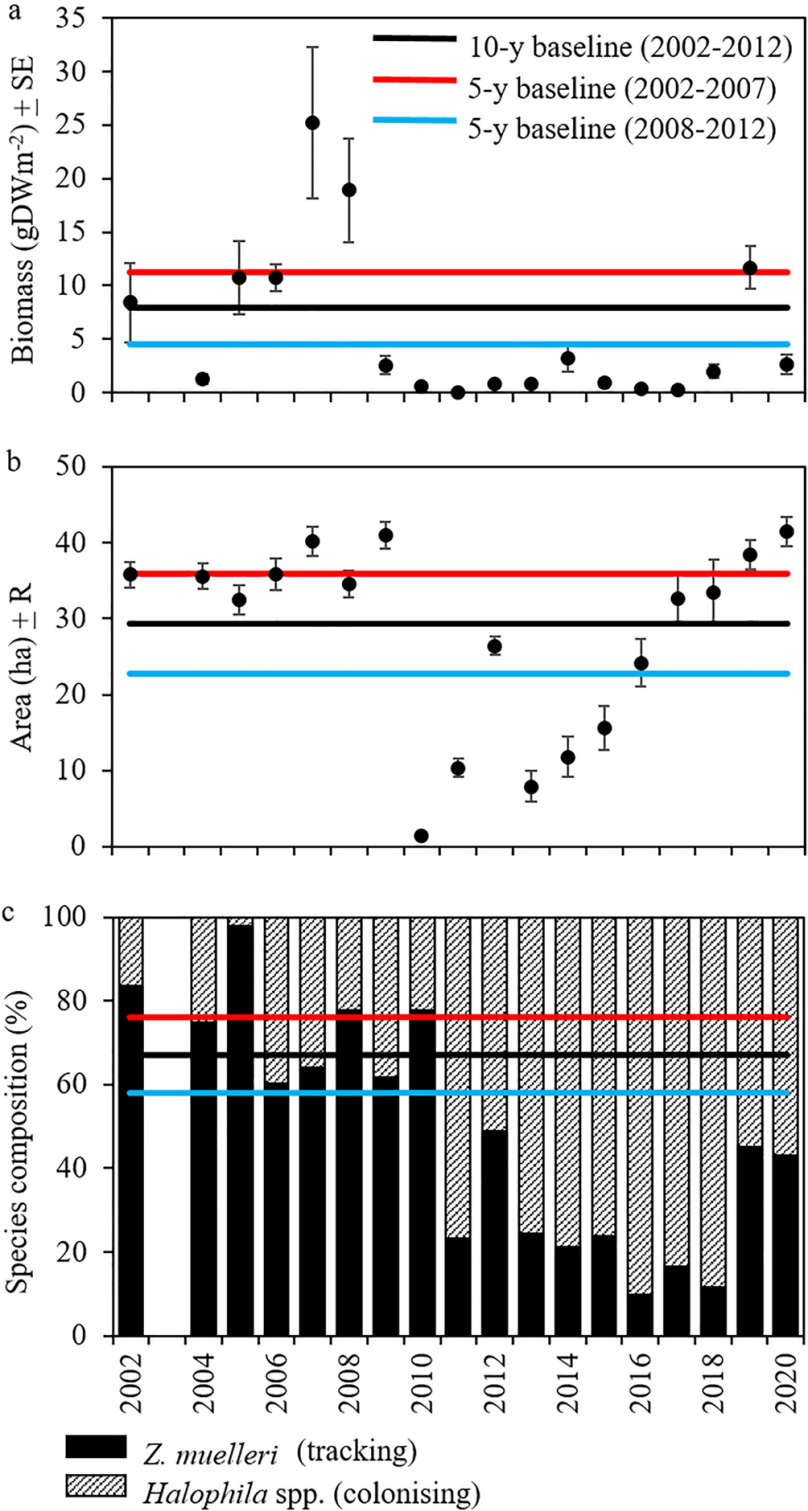
Comparison of baselines for (**a**) biomass (using meadow 104 as an example), (**b**) area (meadow 4), and (**c**) species composition (meadow 8) calculated using 10 years of data (black line), 5 years (2002–2007) during *El Niño* conditions (red line), and 5 years (2008–2012) that included several years of intense *La Niña* conditions (blue line). Species composition in (**c**) shows baseline for dominant (tracking) species *Zostera muelleri* relative to less persistent, colonising *Halophila* species.

The majority of Gladstone’s seagrass meadows have substantial inter-annual variability in the three indicators^30^. It was important that our baseline (also called benchmark or reference conditions)^31^ for each indicator in each meadow incorporated this variability. This involved establishing an appropriate time period against which to assess annual seagrass condition. We compared three time periods. Shorter timeframes were assessed using 5-year averages from the first five years of monitoring (2002–2007) during *El Niño* conditions and the subsequent 5-years (2008–2012) that included *La Niña* conditions in 2009–2011; these shorter time periods over-estimate or under-estimate baseline values, respectively, due to the cycle of decline and recovery (Fig. 2). We selected the 10-year average because this incorporated a decadal cycle typical of GBRWHA seagrasses, with peaks in seagrass condition typically reported during *El Niño* periods when seagrass growing conditions are ideal (high light, low rainfall) and seagrass condition declines associated with *La Niña* years when rainfall and river flow are above average and benthic light is reduced^18^.

Baseline values for biomass and species composition were calculated using the average of the annual meadow averages (biomass, species composition) of the first 10 years of monitoring data. Annual species composition was calculated as the percent contribution of each species to mean meadow biomass for a given year. This approach ensured that equal weight was given to each year regardless of inter-annual variations in the number of sites surveyed within each meadow (as meadow size varied among years).

**Figure 3:**
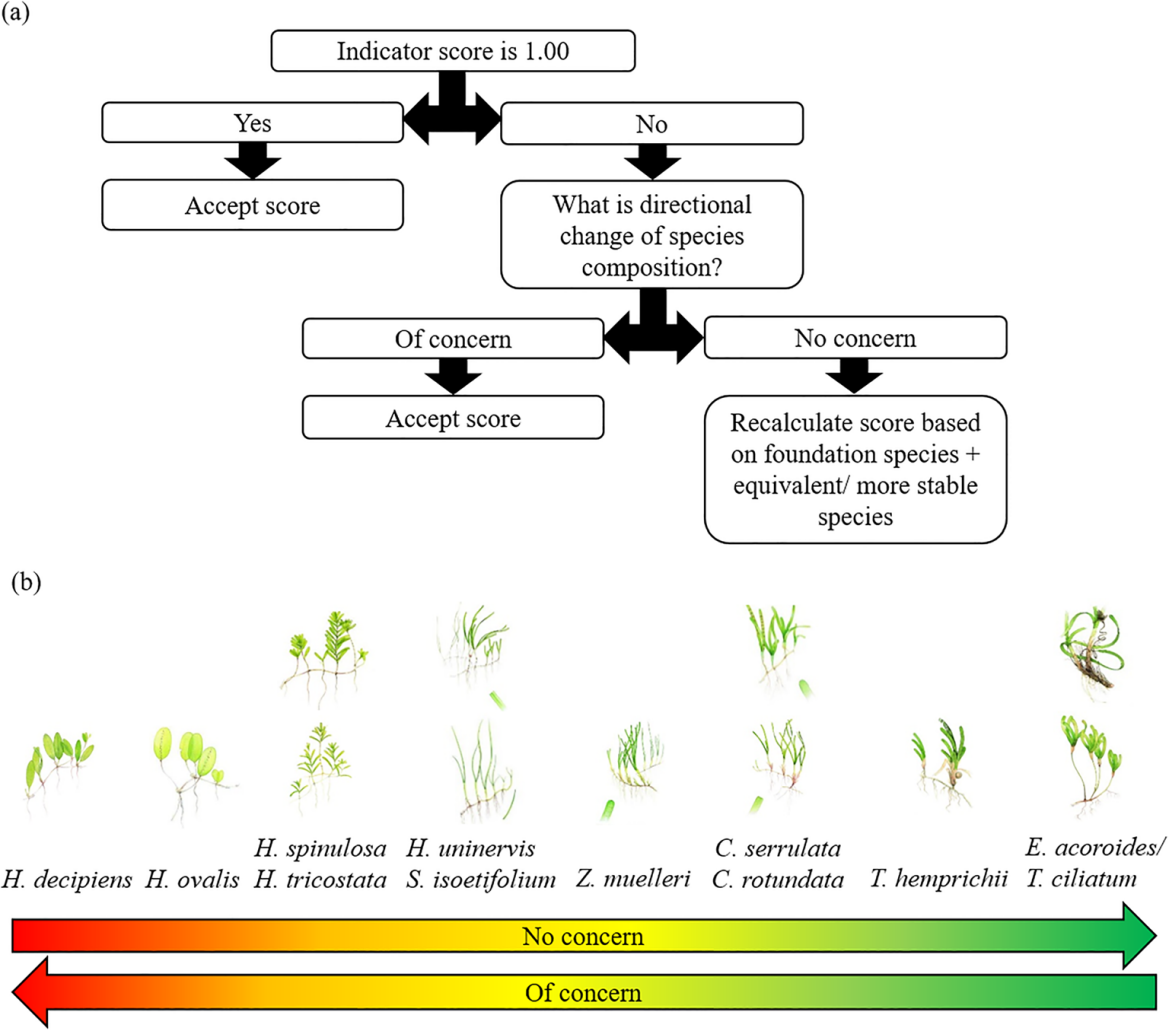
(**a**) Decision tree and (**b**) directional change assessment for grading and scoring seagrass species composition. The decision tree was developed to include all Queensland species so that it could be applied beyond Gladstone Harbour. These species are: *Halophila decipiens*, *Halophila ovalis*, *Halophila spinulosa*, *Halophila tricostata*, *Halodule uninervis*, *Syringodium isoetifolium*, *Zostera muelleri*, *Cymodocea serrulata*, *Cymodocea rotundata*, *Thalassia hemprichii*, *Enhalus acoroides* and *Thalassodendron ciliatum*.

Many of the monitoring meadows have a mix of species. To account for this we classed meadows as single species dominated if one species comprised ≥ 80% of the meadow’s baseline, otherwise meadows were classed as mixed species. Where a meadow baseline contained an approximately equal split in two dominant species, i.e. each species accounted for 40–60% of the baseline, the baseline was set according to the percent composition of the more persistent/stable species (Fig. 3). The dominant and/or more persistent/stable species is referred to as the “tracking” species in species composition assessments.

#### Grades and scores defined

We used a 0–1 score range and score, a report card grade conversion that was mandated by GHHP, and which leads to a typical five-point grading system: A (very good), B (good), C (satisfactory), D (poor) and E (very poor) (Table 1)^6^.

**Table 1:**
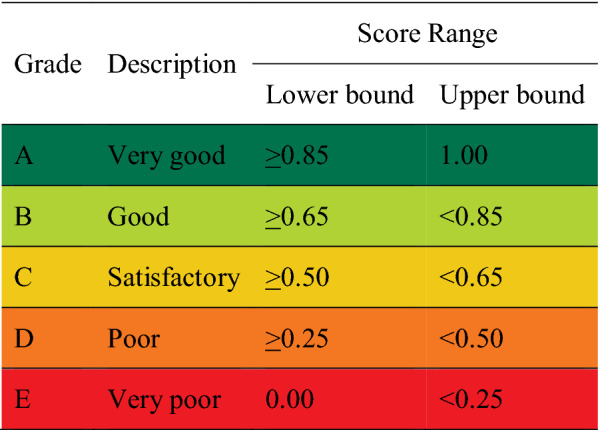
Score range used in five-point grading system for the seagrass report card^6^.

#### Grade thresholds defined

Gladstone Harbour seagrass meadows have differing degrees of variability for each indicator. Meadows can be variable (transitory) or stable (enduring), depending on the species within the meadow and the range and variability of the abiotic environment^32^. We defined the thresholds for grades A-E based on the percent change in biomass, area, or dominant species relative to the 10-year baseline. Narrower grade thresholds were imposed where the baseline of a meadow indicator was defined as stable, because a change in a stable meadow has more ecological relevance than change in a meadow with a history of high variability (Table 2). For each meadow we used the coefficient of variation (CV) to classify species composition and biomass as historically stable (CV: < 40%) or variable (CV: ≥ 40%). Four categories were defined for meadow area due to much higher ranges in CV relative to other indicators: highly stable (CV: < 10%), stable (CV: ≥ 10% < 40%), variable (CV: ≥ 40% < 80%), and highly variable (CV: ≥ 80%) (Table 3). The CV was calculated for each indicator by dividing the standard deviation of the 10 baseline years by the 10-year baseline.

**Table 2:**
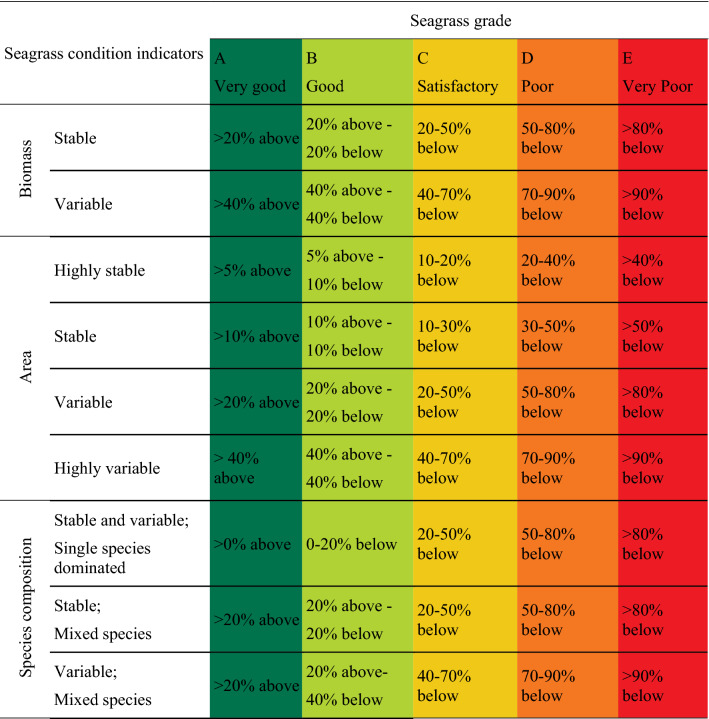
Thresholds used to determine grades and scores for biomass, area and species composition according to indicator stability/variability.

**Table 3:** Classifications representing the historical variability of seagrass condition 10-year baselines for biomass, area and species composition for each monitoring meadow (see Fig. 5 for map of zones).

Meadow ID	Biomass	Area	Species composition (dominant species)
21	Variable	Stable	Stable—mixed species (*Zostera muelleri*)
4	Variable	Variable	Variable—mixed species (*Zostera muelleri*)
5	Variable	Stable	Variable—mixed species (*Zostera muelleri*)
6	Variable	Stable	Variable—mixed species (*Zostera muelleri*)
7	Variable	Highly variable	Stable—single species (*Halophila decipiens*)
8	Variable	Stable	Stable—mixed species (*Zostera muelleri*)
52	Variable	Variable	Variable—mixed species (*Halophila ovalis*)
58	Variable	Highly variable	Variable—mixed species (*Zostera muelleri*)
43	Stable	Highly stable	Stable—single species (*Zostera muelleri*)
48	Variable	Variable	Stable—single species (*Halodule uninervis*)
60	Variable	Variable	Variable—single species (*Zostera muelleri*)
94	Variable	Stable	Stable—single species (*Zostera muelleri*)
96	Variable	Variable	Stable—single species (*Zostera muelleri*)
104	Variable	Stable	Stable—single species (*Zostera muelleri*)

### Annual assessments of seagrass condition

#### Annual grades and scores

Annual seagrass condition was determined by assessing each meadow’s area, biomass and species composition in a given year relative to that meadow’s baseline, stability classification and thresholds (Tables 2 and 3). Annual scores were calculated by scaling annual area, mean biomass, or mean species composition against the score range for that grade (see Appendix S1 in Supporting Information for an example of meadow area score calculations). Scaling was required because the score range in each grade was not equal, ranging from 0.25 (e.g. very poor) to 0.15 (very good) (Table 1). For species composition the upper limit for the very good grade (score = 1.00) was set as 100% (as a species could never account for > 100% of species composition). For biomass and meadow area the upper limit was set as the year with the maximum mean plus standard error (i.e. the top of the error bar), compared among years during the 10-year baseline period.

We developed a decision tree to determine whether a change in the composition of the tracking species represents a decline or improvement in species condition (Fig. 3). Seagrass species life history strategies can be described along a gradient from colonising (fast shoot turnover, dormant seeds, low physiological resistance, rapid ability to recover), to persistent (slow shoot turnover, no seed dormancy, high physiological resistance, slow ability to recover)^32^. Seagrass species were ranked using a modified Kilminster et al.^32^ model with adjustments made for Queensland conditions, such as ranking *Halophila* by species in recognition of their different responses to environmental conditions such as benthic light^33^. Where an annual assessment of species composition was scored less than 1.00 (i.e. the tracking species did not contribute 100% to mean meadow biomass), an assessment was made whether the species composition had declined or improved. If a decline in meadow condition was indicated with a shift to more colonising species, e.g. a decline in *Z. muelleri* relative to *H. ovalis*, then the score would be maintained (see Fig. 3c for an example of this scenario). If the alternative scenario occurs and the tracking species had declined relative to an equivalent or more persistent species, the species composition score and grade for that year would be recalculated to include those additional species (Fig. 3).

#### Score aggregation

Biomass, area and species composition indicators were aggregated to provide (1) an overall meadow condition, (2) the seagrass condition in each GHHP zone and (3) a seagrass condition for all of Gladstone Harbour. Overall meadow condition was recorded as the lowest indicator score for either biomass or area. The lowest score, rather than the mean of the three indicator scores, was applied in recognition that a poor grade for either of these indicators described a seagrass meadow in poor condition. This method allows the most conservative estimate of meadow condition to be made^34^ and reduces the potential for problematic results to be diluted when averaged^35^. Where species composition was the lowest score, it was recorded as contributing 50% to the overall meadow score with the next lowest indicator score (area or biomass) contributing the remaining 50%. This weighting was applied to prevent a meadow receiving a zero score due to species composition changing despite having measurable area and biomass of less persistent species. This weighting acknowledges that species composition is an important characteristic of a seagrass meadow in terms of defining meadow stability, resilience and ecosystem services, but is not as fundamental as having some seagrass present, regardless of species, when defining overall condition. All final Gladstone Harbour zone grades/scores were calculated by averaging the overall meadow scores for each monitoring meadow within a given zone. Combining meadow scores into zone scores ensured the Gladstone Harbour score was not weighted by uneven sampling effort among zones. The annual Gladstone Harbour grade/score is the average of each zone score.

### Application in 2020 report card

**Figure 4:**
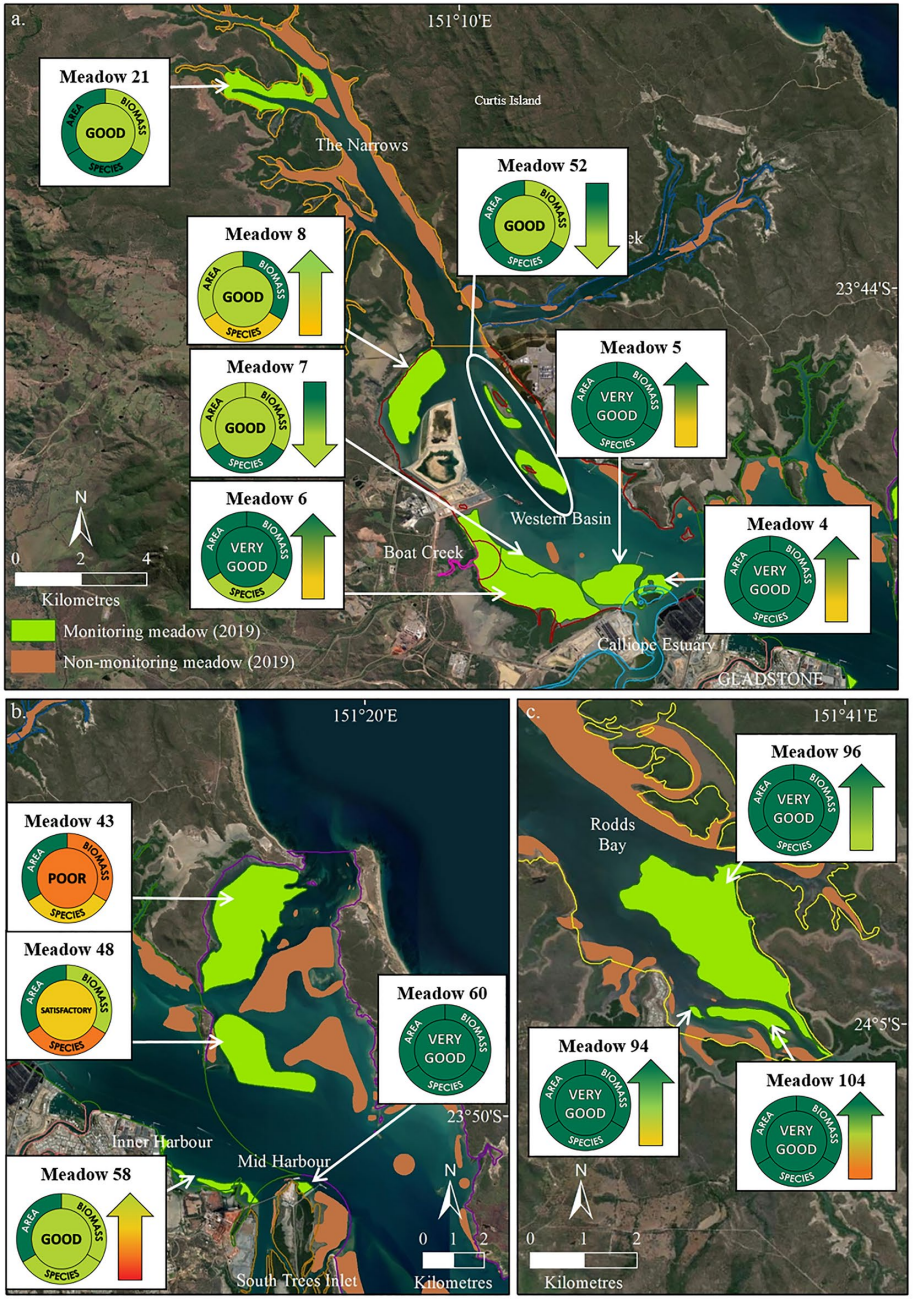
Seagrass condition for each indicator and overall meadow condition for 14 monitoring meadows within Gladstone Harbour Zones in 2020 report card (November 2019 field survey). (**a**) The Narrows, Graham Creek and Western Basin, (**b**) Inner Harbour, Mid Harbour and South Trees Inlet, and (**c**) Rodds Bay. Changes in overall meadow condition relative to the previous year’s report card are described using arrows; an “up” arrow indicates an improved grade change; a “down” arrow indicates a decline in grade; no arrow indicates the grade is unchanged (stable). Map created using ArcGIS software version 10.8 by Esri (www.esri.com). Satellite image copyright Esri.

To demonstrate the results of implementing these methods we use the 2020 Gladstone reporting year as an example (field data collected in late 2019 during the seagrass growing season). Seagrass condition in Gladstone Harbour in the 2020 reporting year was good or very good in five of six monitoring zones, as was overall meadow condition in 12 of the 14 meadows within those zones (Table 4). Seagrass meadows in the Mid Harbour Zone were in poor condition due to low biomass in Meadow 43 and a reduction in the tracking species *Z. muelleri* relative to more colonising species at Meadows 43 and 48 (Table 4; Fig. 4).

**Table 4:**
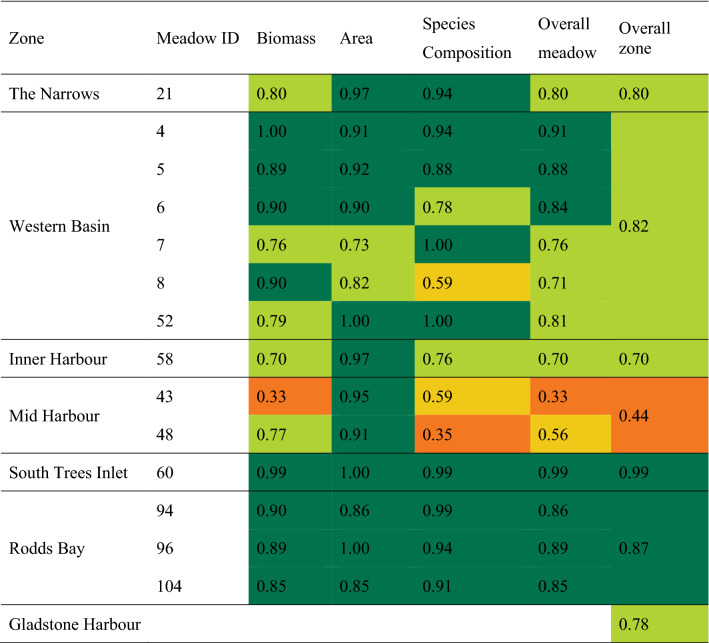
Grades and scores for seagrass indicators (biomass, area and species composition), overall meadow, zone, and Gladstone Harbour scores for the 2020 report card (2019 field survey).

**Figure 5:**
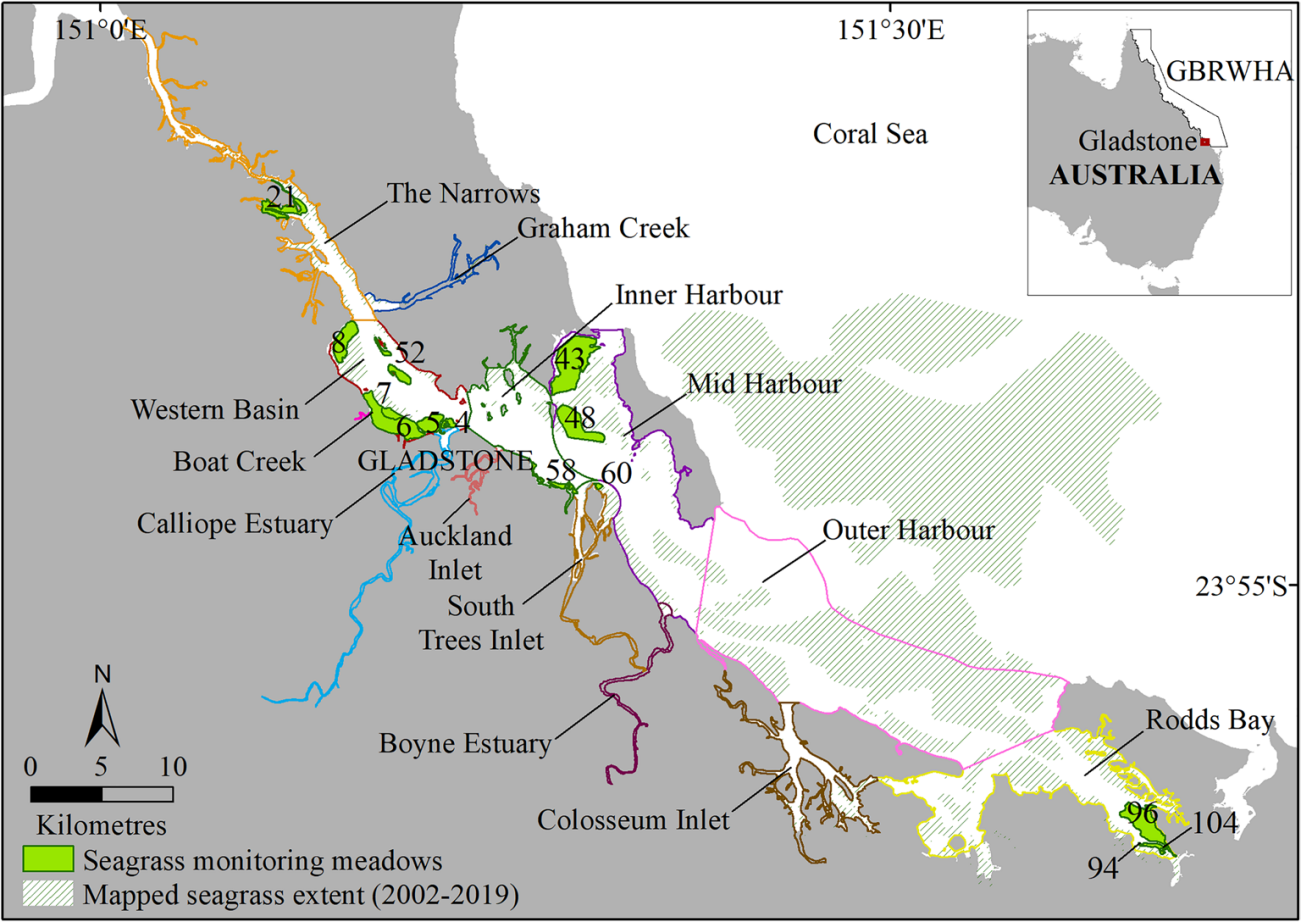
Gladstone Harbour with 13 Gladstone Healthy Harbour Partnership zones, 14 seagrass monitoring meadows surveyed in November 2019 (2020 GHHP reporting year), and composite of seagrass extent mapped approximately every 3–5 years between 2002 and 2019. Inset map: Survey area and Great Barrier Reef World Heritage Area (GBRWHA). Map created using ArcGIS software version 10.8 by Esri (www.esri.com).

Cells are coloured according to grade (see Table 1 for grading scale and colour legend and Fig. 5 for map of zones).

Seagrass was in the best overall condition in the 2020 report card since seagrass loss associated with tropical cyclones and flooding in 2009–2011 (see Appendix S2 in Supporting Information). Nine meadows improved to pre-2010 conditions and a record biomass and/or area was recorded for five monitoring meadows. Improved seagrass condition occurred among different seagrass meadow types and reporting zones, including The Narrows, Western Basin, Inner Harbour and Rodds Bay (Fig. 4).

## Discussion

Seagrass is an ideal indicator for monitoring marine environmental trends due to its sensitivity to environmental conditions and its importance to coastal and nearshore marine ecosystems^4,16,17^. In this report card we address key challenges in reporting condition for seagrass communities typical of tropical and subtropical regions. This includes implementing different thresholds to deal with high spatial and temporal variability for transitory and opportunistic species, developing a decision tree to deal with species changes in diverse communities, and allowing for varied responses in seagrass condition indicators among meadows growing under different environmental conditions within the same region. Assigning indicator scores at the meadow level according to meadow-specific parameters and targets, then combining spatially to aggregate information for management zones relevant to coastal stakeholders, provides a powerful means to understand the status of seagrass meadows at a regional scale. There have now been seven consecutive years (2014–2020) of reporting seagrass condition in a report card format for Gladstone Harbour and the results indicate our approach is effective in quantifying changes over time.

Our selection of species composition, above-ground biomass and meadow area are indicators that represent seagrass as a diverse habitat; recognize the critical role seagrass plays in providing food and habitat for important species including dugong, turtle, and economically important fishery species; and include the physical benefits seagrass meadows provide such as sediment stabilisation and filtration. The inclusion of indicators common to other seagrass monitoring programs and where field data collection is relatively straightforward, is not overly destructive to the plant and does not incur the expense of lab-based processing for other physiological and biochemical indicators, means there is a high likelihood that these metrics will continue to be collected and data are comparable among locations. We considered response times to degradation and recovery and avoided indices likely to be highly stochastic on short-term timeframes, e.g. days-weeks^4^. Our approach here is a reporting approach; relatively simple, easily replicated by non-scientific citizens groups while avoiding complex modelling used to provide a more detailed evaluation of environmental management performance^9,18^.

In multi-species seagrass meadows shifts in species are a key indicator of disturbance, with well-documented cases of post disturbance species successions, including within the GBRWHA^36,37^. These species shifts can occur independently of changes in other meadow scale metrics such as meadow area and biomass but still have important implications for seagrass ecosystem services. Incorporating species composition into the report card in a way that indicates ecological condition was essential to understanding meadow health. Species vary in their morphologies, growth rates, root structures and leaf turnover rates, which influences their capacity to provide important ecosystem services. Changes in species composition influence the role meadows play in coastal ecosystems^38^ and that meadow’s resilience to disturbances^27^. Fish species display a distinct preference for particular seagrasses characterised by different architecture^39^ and shifts in seagrass species composition can lead to changes in the abundance and diversity of fish and other macrofauna such as crabs and shrimp^40^. Stiffness, biomass, density, leaf length and morphology all influence the coastal protection value of seagrasses, with large, long living, slow growing seagrass species affording the greatest protection^41^. Species composition is a known contributor to variability in carbon stocks^42^ with larger bodied species generally associated with higher sedimentary organic carbon stocks. Larger bodied, persistent species generally have a higher physiological resistance to disturbance, while small bodied, colonising species have a rapid ability to recover^32^.

In the years during and immediately after the 2009–2011 *La Niña* the contribution to meadow biomass of the largest and most persistent genera *Zostera* declined relative to the colonising *Halophila* species Fig. 2; Ref.^30^, a typical pattern following environmental impact^32^. Our development of a decision tree to decipher whether a change in species composition signifies an improvement or decline in species condition assists in providing a framework for assessing seagrass condition in multi-species seagrass communities.

The high variability of tropical and subtropical seagrasses highlights the importance of having adequate reference data to appropriately assess seagrass condition in the context of environmental cycles and meadow location. Gladstone Harbour monitoring spanned *El Niño* periods ideal for seagrass growth, and a *La Niña*-associated period in 2009–2011 where frequent tropical cyclones, high rainfall and flooding reduced seagrass biomass, extent and species composition in the harbour and throughout the central and southern GBRWHA^43,44^. Our 10-year baseline period captured this decadal cycle of decline and recovery of seagrass condition. Scaling meadow condition scores on the 0–1 scale was an important step in allowing for standardised comparisons that ensured individual meadows were not penalized because they did not meet unattainable goals, e.g. the absence of *Z. muelleri* in what has historically been a *H. ovalis* meadow, or meadow size being assessed relative to that meadow’s potential extent and not relative to size of neighbouring meadows. We have 17 years of seagrass monitoring data collected with standardized and consistent methods which allowed us to compare approaches to setting baselines and make well-informed decisions when selecting indicators and defining thresholds in developing this report card. Where data or knowledge of a system is limited, we recommend periodic reviews of baseline/benchmark/reference conditions to ensure the report card’s grades and scores are ecologically meaningful and transparency around levels of confidence.

Report cards are designed to integrate available data and provide a snapshot of condition and communicate trends; they do not establish direct relationships between seagrass metrics and other environmental indicators beyond a simple narrative of inference and cross-referencing^27,45^. Some of this detail will be available in background technical reports but it is unlikely that most report card users (e.g. community groups, politicians) will access that level of information. Report cards are not designed to directly assess progress towards environmental goals or to necessarily conclude on the success or otherwise of management programs. That information is outside the scope of report card frameworks and requires a more complex approach and analysis^18^. This emphasises both a limitation of report cards and the importance of carefully designing them to produce scores and grades that are accurate reflections of environmental condition and trends. In this way condition indicators provide an early warning of change and the report card represents just one step in the adaptive management cycle (to assess current conditions and identify potential problems). Adaptive management should aim to halt further decline and return habitats to their desired state^9^. Report card outcomes therefore should link to management actions that will achieve this^35^. The inclusion of seagrass to the GHHP report card adds value by including a habitat indicator suitable for providing an early warning of environmental decline that may not be evident in other habitat indicators (e.g. coral, mangroves) and assessing the effectiveness of management interventions, such as restoration, in the future.

### Regional application

Annual report cards using our approach are now produced throughout north-east Australia, including at Karumba^46^, Weipa^47^, Cairns^48^, Mourilyan^49^, Townsville^50^, Abbot Point^51^, Hay Point^52^ and Clairview^53^. In 2021, the Torres Strait seagrass report card applied our method to integrate condition assessments using data collected from more than 20 seagrass meadows across four separate monitoring programs^54^. Within the GBRWHA, the report card scores produced by our method are integrated with other data sources that use different indicators (e.g. tissue nutrients, percent cover, reproduction; https://www.seagrasswatch.org/marine-monitoring-program/) to create an holistic annual seagrass condition assessment and reporting for regional areas, including the Mackay-Whitsunday-Isaac Healthy Rivers to Reef Partnership (https://healthyriverstoreef.org.au/) and Wet Tropics Waterways (https://wettropicswaterways.org.au/). The successful application of our approach to these locations demonstrates the utility of the method developed and ensures that this critical and diverse marine habitat is increasingly incorporated into environmental assessments and decision making.

Our scoring approach has allowed seagrass condition to be incorporated into more comprehensive environmental condition assessments. The condition of Gladstone Harbour is reported by GHHP across four broad themes—environmental, social, cultural and economic. Environmental reporting is divided into three sub-groups: water and sediment quality, habitats, and fish and crabs, with seagrass representing one of three habitat indicators (plus coral and mangroves) (http://ghhp.org.au/report-cards/2020). In the Mackay-Whitsunday-Isaac Healthy Rivers to Reef Partnership seagrass condition is combined with coral and water quality indicators to deliver inshore marine scores and grades in the annual report card (https://healthyriverstoreef.org.au/). In the Wet Tropics Waterways report card seagrass condition is combined with mangroves, flow, riparian extent and fish barriers to give a habitat and hydrology score, which is then combined with water quality for a broader estuary assessment, while for inshore reporting seagrass scores are combined with water quality and coral (https://wettropicswaterways.org.au/).

## Conclusion

Our report card framework summarises in an easily replicated way large amounts of spatially and temporally complex data into reliable assessments of seagrass condition. The seagrass indicators selected – meadow area, above-ground biomass and species composition represent the key functions and ecosystem services seagrass habitats provide. The grades and scores were calculated using a rigorous approach, but the reporting scheme makes this assessment of seagrass condition accessible to a broad audience in a consistent format that environmental managers and stakeholders can have confidence in. Our case study focuses on Gladstone Harbour, but the approach has been used regionally throughout tropical Queensland with success and has international relevance due to the ease with which it can be applied to other seagrass monitoring locations.

## Methods

### Study area

Gladstone Harbour in Queensland, Australia, is an area of significant environmental and economic importance. The harbour contains a major multi-commodity industrial port that is a key export hub for coal, bauxite, alumina, aluminium, cement and liquefied natural gas, with high economic and industrial value to Australia^55^. The harbour is also within the GBRWHA, adjacent to the Great Barrier Reef Marine Park, and incorporates a Dugong Protection Area and three protected Fish Habitat Areas. These competing values and a public concern with declining environmental and social values^35,45,56,57^ makes Gladstone Harbour an excellent example of the need for adaptive management informed by up-to-date scientifically robust data that is easily communicated to a range of stakeholders.

Seagrass is a major marine habitat in Gladstone Harbour, with up to 29,000 ha mapped in intertidal and subtidal waters (Fig. 5)^30^. Several of the tropical seagrass species found in Gladstone Harbour are transitory, sometimes annual and can rapidly colonise if habitat conditions become suitable. Meadows are commonly multispecies and this, combined with the levels of variability, challenges simplistic approaches to reporting trends.

The Gladstone seagrass report card was developed on request for the Gladstone Healthy Harbour Partnership (GHHP). The GHHP was established in 2013 in response to environmental declines in Gladstone Harbour with the objective of improving decision making around environmental management and the reporting of change in environmental metrics^57^. The GHHP includes representatives of industry, government, traditional owners, scientists and the local community that support research, monitoring and reporting on the environmental, social, cultural and economic health of the harbour (https://www.ghhp.org.au/what-we-measure). The report card’s main function is to present an annual health assessment of Gladstone Harbour and explanation of trends over time to a general audience; reporting occurs for 13 Gladstone harbour zones (Fig. 5; http://ghhp.org.au/)^35^.

### Historic monitoring data

The first detailed survey of Gladstone Harbour seagrass was conducted in 2002 and a subset of seagrass meadows chosen for monitoring have been assessed annually since 2004^30^, while harbour-scale surveys of all meadows are conducted every 3–5 years (Fig. 5).

Gladstone Harbour monitoring meadows have five seagrass species: *Halophila decipiens*, *Halophila ovalis*, *Halophila spinulosa*, *Halodule uninervis* and *Zostera muelleri* subsp. *capricorni* (abbreviated to *Z. muelleri* for this paper). Twelve monitoring meadows are intertidal and dominated by various species combinations including *Z. muelleri* with a mix of *H. ovalis*, *H. decipiens* and *H. uninervis,* and *H. ovalis* only (meadow 52). Two subtidal meadows monitored are dominated by *H. decipiens* (meadow 7) and *H. uninervis* (meadow 48). The size of the 14 monitoring meadows varies from small (max area < 20 ha; meadows 94, 60) to large (max area up to 500 ha; meadows 6, 43) and encompass a range of seagrass biomass from low (max recorded < 2 g DW m^−2^ ; meadows 4, 6, 7, 8, 52) to high (> 18 g DW m^−2^ ; meadow 43). Gladstone Harbour has two weather seasons, a wet season (late summer—autumn) and dry season (winter—early summer). The seagrasses have a growing period that coincides with the dry season, when seagrasses increase in biomass and area in response to favourable conditions. During the wet season seagrass are senescent and rely on below-ground energy stores or seeds to endure the wet season conditions of flooding, poor water quality and light reductions^15^. We limited the influence of seasonal variation in seagrass growth by only including survey data collected during the peak of the growing season (September–December)^28,58^.

Annual monitoring data was collected using standardised seagrass survey methods for Gladstone Harbour^30^. Seagrass data was collected from haphazardly placed sites each of ~ 10 m^2^. Each site was surveyed using helicopter in a low hover (< 1 m) for intertidal areas and for subtidal areas using boat-based divers, video camera drops and/or van Veen grabs. At each site, latitude, longitude, sediment type and seagrass species presence/absence and biomass were recorded. For each site seagrass above-ground biomass was determined using the “visual estimates of biomass” technique^59,60^ from three replicate 0.25 m^2^ quadrats (helicopter, diving and camera drop sites). For each quadrat an observer assigned a biomass rank, made in reference to a series of ~ 12 quadrat photographs of similar seagrass habitats for which the above-ground biomass had previously been measured. The percent contribution of each seagrass species to above-ground biomass within each quadrat was also recorded. At the survey’s completion, the observer ranked a series of calibration quadrat photographs representative of the range of seagrass biomass and species composition observed during the survey. These calibration quadrats had previously been harvested and the above-ground biomass weighed in the laboratory. A separate regression of ranks and biomass from the calibration quadrats was generated for each observer and applied to the biomass ranks recorded in the field. Field biomass ranks were converted into above-ground biomass estimates in grams dry weight per square metre (g DW m^−2^) for each of the three replicate quadrats at a site. Site biomass and the biomass of each species, is the mean of the replicates. Seagrass biomass could not be determined from sites sampled by van Veen grab, but seagrass presence/absence and species composition was recorded from three replicate grabs with an area of 0.0625 m^2^ at each site.

Seagrass meadows are defined as an accumulation of seagrass plants over a mappable area^61^. Meadow boundaries were mapped using GPS in the helicopter for intertidal meadows and for subtidal meadows estimated using GPS located seagrass presence/absence site data, rectified colour satellite imagery of Gladstone Harbour (source: ESRI), field notes and photographs taken during each survey. A mapping precision estimate for a meadow’s boundaries (in metres) was made for each meadow based on the mapping method used and ranged from < 5 m for intertidal seagrass meadows with boundaries mapped by helicopter to ± 50 m for subtidal meadows with boundaries mapped by distance between sites with and without seagrass. Precision estimates were used to calculate a buffer around each meadow. The area of this buffer is expressed as a meadow’s reliability estimate (R) in hectares. The area of each meadow and R was determined using the calculate geometry function. All spatial analysis was conducted using ArcMap^®^^30^.

## Supplementary Information

Supplementary Information.

## Data Availability

The dataset analysed during the current study is available via request through the Gladstone Healthy Harbour Partnership e-Portal at http://data.ghhp.org.au/.
