# Supplementary material for: A report card approach to describe temporal and spatial trends in parameters for coastal seagrass habitats
Source: Sci Rep. 2023 Feb 9;13:2295. doi: 10.1038/s41598-023-29147-1 (PMC9911721; doi:10.1038/s41598-023-29147-1)

Supporting Information

Appendix S1. An example of calculating a meadow score for area in satisfactory condition in 2020

1. Determine the grade for the 2020 (current) area value (i.e. satisfactory).
2. Calculate the difference in area (A_diff_) between the 2020 area value (A_2020_) and the area value of the lower threshold boundary for the satisfactory grade (A_satisfactory_):

$A_{\mathrm{diff}}= A_{2020}-A_{\mathrm{satisfactory}}$

Where A_satisfactory_ or any other threshold boundary will differ for each condition indicator depending on the baseline value, meadow class (highly stable [area only], stable, variable, highly variable [area only]), and whether the meadow is dominated by a single species or mixed species.

1. Calculate the range for area values (A_range_) in that grade:

$A_{\mathrm{range}}= A_{\mathrm{good}}-A_{\mathrm{satisfactory}}$

Where A_satisfactory_ is the upper threshold boundary for the satisfactory grade.

Note: For species composition, the upper limit for the very good grade is set as 100%. For area and biomass, the upper limit for the very good grade is set as the maximum value of the mean plus the standard error (i.e. the top of the error bar) for a given year during the baseline period for that indicator and meadow.

1. Calculate the proportion of the satisfactory grade (A_prop_) that A_2020_ takes up:

$$A_{\mathrm{prop}}= \frac{A_{\mathrm{diff}}}{A_{\mathrm{range}}}$$

2. Determine the area score for 2020 (Score_2020_) by scaling A_prop_ against the score range (SR) for the satisfactory grade (SR_satisfactory_), i.e. 0.15 units:

Where LB_satisfactory_ is the defined lower bound (LB) score threshold for the satisfactory grade, i.e. 0.50 units.

**Appendix S2.**

Table S1. Overall scores and grades for individual monitoring meadows, 2002 – 2019 field surveys (2003 – 2020 GHHP reporting years). No survey conducted in 2003 (2004 reporting year). Grades: Very good, dark green; good, light green; satisfactory, yellow; poor, orange; very poor, red. See Table 1 in manuscript for grades that correspond with colours below


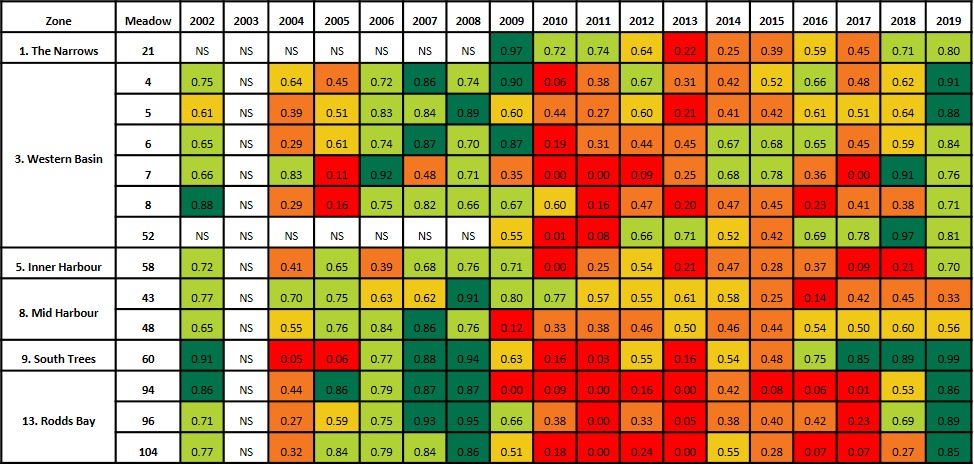

Supplement: Supplementary file 1 — Supplementary Information. [file 41598_2023_29147_MOESM1_ESM.docx]
